# CAMSAP1 Mutation Correlates With Improved Prognosis in Small Cell Lung Cancer Patients Treated With Platinum-Based Chemotherapy

**DOI:** 10.3389/fcell.2021.770811

**Published:** 2022-01-11

**Authors:** Yonglin Yi, Zhengang Qiu, Zifu Yao, Anqi Lin, Yimin Qin, Ruizhan Sha, Ting Wei, Yanru Wang, Quan Cheng, Jian Zhang, Peng Luo, Weitao Shen

**Affiliations:** ^1^ Department of Oncology, Zhujiang Hospital, Southern Medical University, Guangzhou, China; ^2^ Department of Oncology, First Affiliated Hospital of Gannan Medical University, Guangzhou, China; ^3^ The First Clinical Medical School, Southern Medical University, Guangzhou, China; ^4^ Department of Neurosurgery, Xiangya Hospital, Center South University, Changsha, China

**Keywords:** small cell lung cancer, CAMSAP1, platinum-based chemotherapy, drug sensitivity, biomarker

## Abstract

Platinum-based chemotherapy is the first-line treatment for small cell lung cancer (SCLC). However, due to patients developing a resistance to the drug, most experience relapse and their cancer can become untreatable. A large number of recent studies have found that platinum drug sensitivity of various cancers is affected by specific gene mutations, and so with this study, we attempted to find an effective genetic biomarker in SCLC patients that indicates their sensitivity to platinum-based drugs. To do this, we first analyzed whole exome sequencing (WES) and clinical data from two cohorts to find gene mutations related to the prognosis and to the platinum drug sensitivity of SCLC patients. The cohorts used were the Zhujiang cohort (N = 138) and the cohort reported by George et al. (N = 101). We then carried out gene set variation analysis (GSVA) and gene set enrichment analysis (GSEA) to investigate possible molecular mechanisms through which these gene mutations affect patient prognosis and platinum drug sensitivity. We found that for SCLC patients, CAMSAP1 mutation can activate anti-tumor immunity, mediate tumor cell apoptosis, inhibit epithelial-mesenchymal transition (EMT), improve prognosis, and improve platinum drug sensitivity, suggesting that CAMSAP1 mutation may be a potential biomarker indicating platinum drug sensitivity and patient prognosis in SCLC.

## Introduction

Lung cancer is one of the most common malignant tumors in the world, and it is divided into two categories: small cell lung cancer and non-small cell lung cancer. Small cell lung cancer (SCLC) accounts for 13–15% of all lung cancers and has a high degree of malignancy, having a 5-year survival rate of less than 7% (Govindan et al., 2006; Sabari et al., 2017; Farago and Keane, 2018; Tsoukalas et al., 2018; Qiu et al., 2019a; Li et al., 2020). In 70% of SCLC patients, extended-stage SCLC (ES-SCLC) was diagnosed. Platinum-based chemotherapy is the first-line treatment of ES-SCLC with an effective rate of 50–75% in the initial stage of treatment (Rossi et al., 2012; Waqar and Morgensztern, 2017). However, most patients relapse within 6 months due to the development of drug resistance, leading to disease progression and in some cases, death (Sabari et al., 2017). Topotecan is the only second-line drug certified by the FDA for the treatment of ES-SCLC, and is mainly effective in the patients who are also sensitive to first-line treatment (Ardizzoni et al., 1997).

The development of chemotherapy resistance occurs through many mechanisms, such as a decrease in drug accumulation and apoptosis, an increase in drug inactivation, cell protective autophagy, and the number of cancer stem cells (Hermann et al., 2007; Mani et al., 2015; Luo et al., 2017; Maji et al., 2018; Wang et al., 2018; Qiu et al., 2019b). The number of cancer stem cells is closely related to epithelial-mesenchymal transition (EMT) and the immune microenvironment (Ahmed et al., 2018; Li et al., 2019). Additionally, the activation of anti-tumor immune activity has been shown to reverse chemotherapy resistance by increasing the rate of apoptosis in tumor cells (Xu et al., 2016). Therefore, to improve the prognosis of patients with SCLC, it is very important to find effective biomarkers indicative of platinum drug sensitivity and to explore the mechanisms that affect platinum drug sensitivity.

In recent years, a large number of studies have found that gene mutations can affect the platinum drug sensitivity of various cancers. For example, it has been shown that the overexpression of CCDC69 can activate the p14 ARF/MDM2/p53 pathway in ovarian cancer and that it is associated with a higher sensitivity to platinum drugs (Cui et al., 2019). Also, Qiang Li found that ERCC2 mutation can abrogate nuclear error repair in bladder cancer, thus increasing platinum drug sensitivity (Li Q. et al., 2019). In one more example, I. Lohse was able to show that BRCA1, BRCA2 mutations can improve platinum drug sensitivity of pancreatic cancer via accumulation of DNA damage (Lohse et al., 2015). Despite this wealth of research, the relationship between gene mutations and sensitivity to platinum drugs in SCLC is not clear. Therefore, with this study we explored the mutant genes from two SCLC cohorts in order to determine whether they could be used as biomarkers that indicate platinum drug sensitivity and patient prognosis.

Calmodulin-regulated spectrin-associated protein1 (CAMSAP1), is a member of CAMSAP/Patronin/Nezha family (Hendershott and Vale, 2014). This family localizes on the microtubule minus-end, promoting microtubule stability (Chuang et al., 2014; Hendershott and Vale, 2014; Yau et al., 2014; Gong et al., 2018). The CKK domain (DUF1781) is in the C-terminal of proteins of CAMSAP family, which binds microtubules (Baines et al., 2009) and mediates the association of CAMSAPs with microtubule minus-end (Gong et al., 2018). The CH domain is in the N-terminus of CAMSAPs and is involved in the regulation of actin dynamics CAMSAPs (Baines et al., 2009; Gong et al., 2018). CAMSAP1 is associated with better prognosis in acute lymphoblastic leukemia (Wang et al., 2015). However, there is no report about the role of CAMSAP1 in SCLC.

In this study, whole exome sequencing (WES) data and clinical information from a reported SCLC cohort (reported by George et al., (2015) and an SCLC cohort from Zhujiang Hospital and Sun Yat-sen University Cancer Center, were used to explore the relationships between specific gene mutations, improved prognosis, and increased platinum-based chemotherapy sensitivity. In addition, we also attempted to identify the related mechanisms and potential drugs. Our results show that CAMSAP1 mutation can be used as a prognostic marker in SCLC patients undergoing treatment with platinum-based chemotherapy. CAMSAP1 mutation was associated with better overall survival (OS), and it shows the potential to increase platinum drug sensitivity in patients with SCLC. Analyses of the mechanism showed that CAMSAP1 mutation can activate anti-tumor immunity, mediate apoptosis of tumor cells and inhibit EMT. This study proves that CAMSAP1 mutation can play an important role in predicting prognosis and platinum-based chemotherapy sensitivity in patients with SCLC.

## Methods

### Clinical Cohort and Expression Data

To assess the relationship between gene mutations and platinum drug sensitivity of SCLC, we collected clinical and WES data from two cohorts. The Zhujiang cohort included 138 SCLC patients who were treated with platinum-based chemotherapy in Zhujiang hospital and Sun Yat-Sen University Cancer Center. The reported cohort was downloaded from George’s study and included 101 SCLC patients who were treated with platinum-based chemotherapy. In addition, we obtained the gene expression file for patients in the Zhujiang cohort.

### Identification of Survival-Related Genes

**FIGURE 1:**
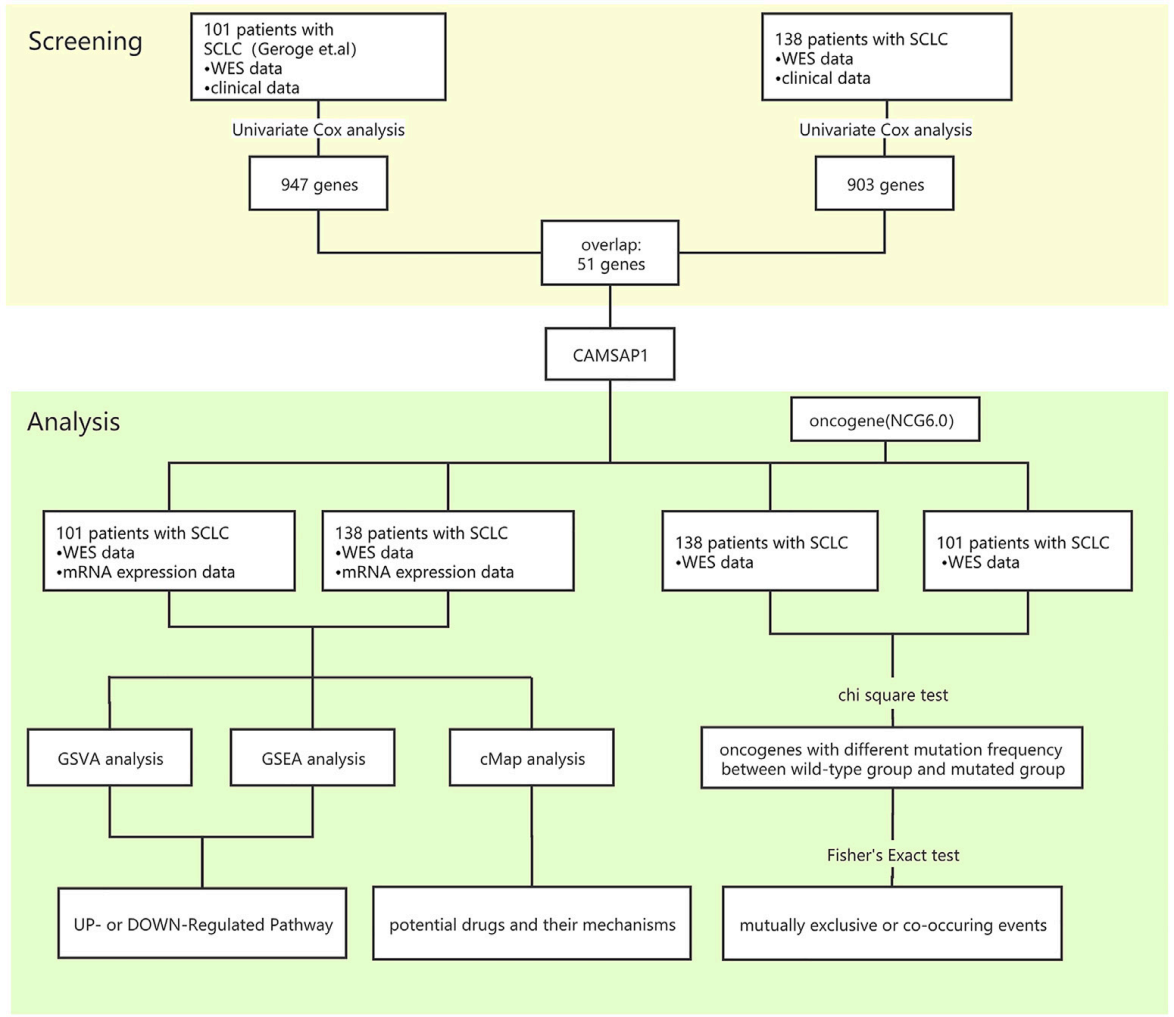
Bioinformatics analysis process. SCLC, small-cell lung cancer; GSVA, gene set variation analysis; GSEA, gene set enrichment analysis; cMAP, connectivity map.

Genes needed to meet the following conditions to be considered survival-related genes in SCLC: 1) the relationship identified by univariate Cox proportional hazards analysis between gene mutation and OS was statistically significant (*p* < 0.05); 2) the frequency of the gene mutation was >5%, and; 3) the common genes were identified in both the Zhujiang cohort and the reported cohort (Figure 1).

### The Mutation Status of Oncogenes and the Relationship Between Gene Mutations

The NCG6.0 database (http://ncg.kcl.ac.uk/) consists of protein, expression, and functional data for 2,372 cancer genes. We used this database to assist in screening out oncogene mutations from the two cohorts. The R package “maftools” was used to identify mutual exclusivity and co-occurrence among gene mutations.

### Enrichment Analysis of Differentially Expressed Genes and Pathways

The R package “limma” was used to identify differentially expressed genes (DEGs) in the CAMSAP1-MT group and CAMSAP1-WT group. The calculation formula of the LogFC cut-off value is:

$$\left|\bar{\mathrm{LogFC}}\right|\mathrm{+2*\sigma}\left(\left|\mathrm{LogFC}\right|\right)$$

The LogFC cut-off value was 0.766 in the Zhujiang cohort and 0.723 in the reported cohort. We used heatmaps to show the expression of the top 20 DEGs with the largest fold change (FC) between the CAMSAP1-MT and CAMSAP1-WT groups, and volcano plots to visualize the DEGs. Using the C2 collection (curated gene sets) obtained from the Molecular Signatures Database of the Broad Institute (MSigDB), we calculated the GSVA score for each patient using the R package “GSVA”. Patients were divided into high- and low-GSVA score groups according to the median GSVA score, and a Kaplan-Meier survival analysis was performed on the two groups. Enrichment analysis of gene annotation (GSEA) was performed by the R package “ClusterProfiler”. When comparing pathway differences in the C2 collection (curated gene sets), *p* < 0.05 was considered to be significant. Then we selected the cancer-associated pathways using PubMed dataset (https://pubmed.ncbi.nlm.nih.gov/).

### Identification of Potential Drugs

In order to identify potential small molecule drugs which target CAMSAP1, we used Connectivity Map (cMAP) database, which is a library of expression files of cell lines treated with different small molecule drugs. We divided the DEGs of the two cohorts into up- and down-regulation groups and entered them into the cMAP online tool (https://portals.broadinstitute.org/cmap/) to obtain a permutation result. cMAP tools (https://clue.io/) were then used to explore the mode-of-action (MoA).

### Statistical Analysis

The relationship between gene mutation frequency and CAMSAP1 status was determined by chi-square test. The relationships between gender, GSVA score and CAMSAP1 status were also determined by chi-square test. To determine the relationship between enrichment score and CAMSAP1 status, permutation test was used, and for the relationships between OS, progression-free survival (PFS) and CAMSAP1 status log-rank test was used. The R package “survival” and “survminer” were utilized to conduct Kaplan–Meier survival analyses, and the relationships between OS, PFS, clinical characteristics and gene mutations were determined by univariate/multivariate cox proportional hazards analysis. Co-occurrence or mutual exclusivity events among gene mutations were identified by Fisher’s Exact test. For all tests, *p* < 0.05 was considered to be statistically significant. All statistical analysis was conducted by R software (version 4.1), and the R package “maftools” was used to analyze and visualize the MAF files.

## Results

### CAMSAP1 Is a Prognostic Marker for SCLC Patients Receiving Platinum-Based Chemotherapy

**FIGURE 2:**
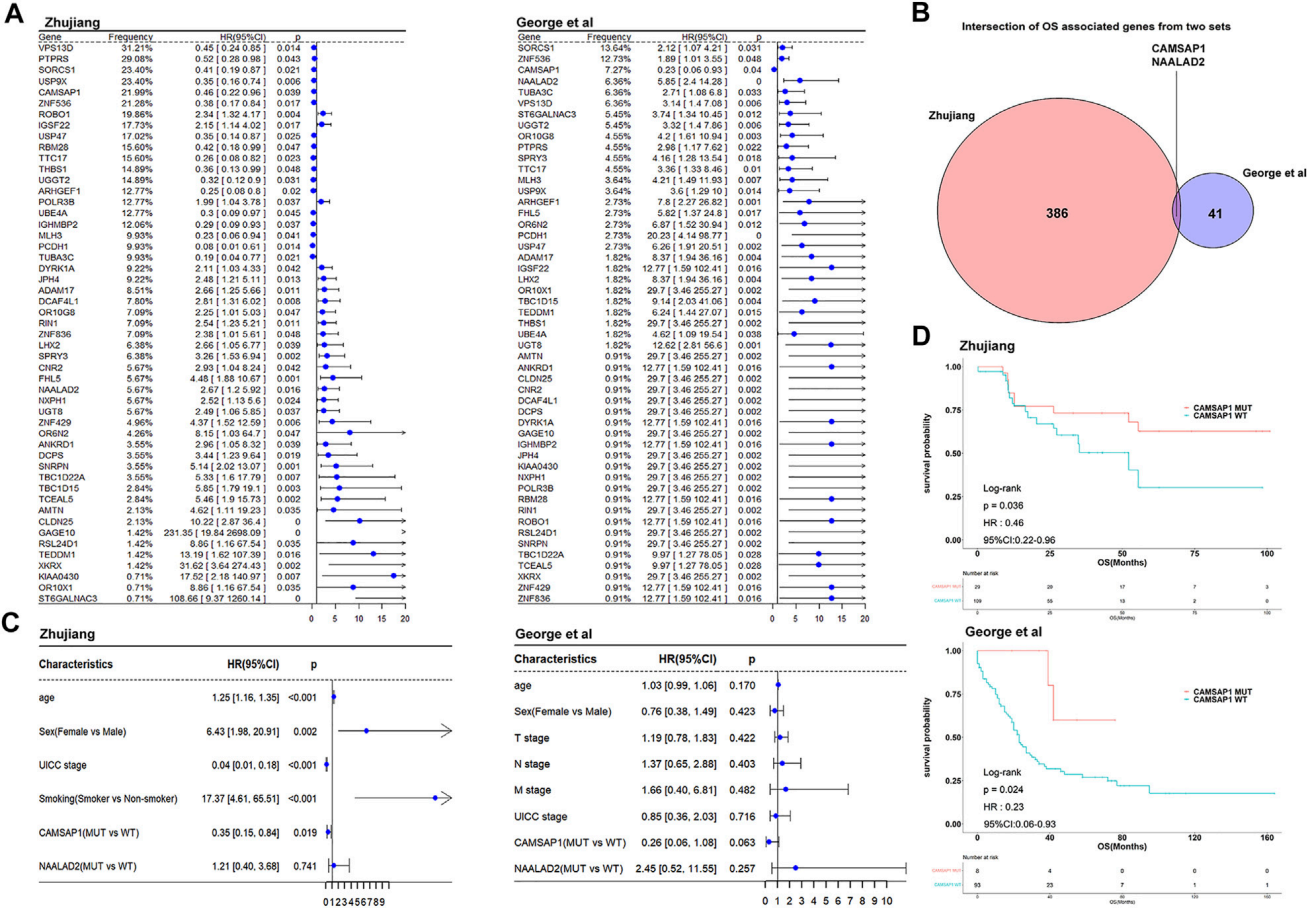
Cox proportional risk regression analysis identifying survival-related genes. **(A)** The forest plots show the univariate cox analysis results of 51 common OS-related gene mutations in the Zhujiang and reported cohorts **(B)** Venn diagram showing the overlap of OS-related genes with mutation frequency >5% in the Zhujiang and reported cohorts. **(C)** Multivariate cox analysis results of gene mutations, clinical factors and OS in the Zhujiang and reported cohorts. **(D)** Kaplan-Meier survival analysis results of OS comparing the CAMSAP1-MT group (red) and the CAMSAP1-WT group (blue) in the Zhujiang and reported cohorts. OS, overall survival; HR, hazard ratio; CL, confidence interval.

We used clinical and WES data from two cohorts of SCLC patients receiving platinum-based chemotherapy treatment. There were 138 patients in the Zhujiang cohort and 101 patients in the reported cohort. For both of these cohorts, no statistical difference in clinical characteristics between CAMSAP1-MT and CAMSAP1-WT groups was found (Supplementary Tables S1, S2). We used univariate cox analysis to explore the effect of gene mutations on OS and there were 51 common gene mutations of statistical significance in two cohorts (*p* < 0.05, Figure 2A). After application of the condition that gene mutation frequency must be more than 5%, there were 386 and 41 OS-related genes in the Zhujiang and reported cohorts respectively. CAMSAP1 and NAALAD2 were shared in both cohorts (Figure 2B). The mutation frequency of CAMSAP1 is 21.99% in Zhujiang cohort and 7.27% in the reported cohort. The results of our analysis showed that CAMSAP1 mutation was associated with better prognosis and NAALAD2 mutation was associated with worse prognosis. The univariate cox statistical index (*p* < 0.05) was introduced into multivariate cox. The results showed that in the Zhujiang cohort age, sex, UICC stage, smoking and CAMSAP1 mutation were independent factors affecting prognosis, and that no significant relationship between NAALAD2 and OS exists (HR = 1.21, 95%Cl 0.40–3.68, *p* = 0.741). In the reported cohort, there was no independent factor affecting prognosis found (Figure 2C). Finally, we selected CAMSAP1 as a candidate molecule for a mutation related to increased drug sensitivity and better prognosis in both cohorts. To further explore the predictive function of CAMSAP1, we performed a Kaplan-Meier survival analysis that showed that patients in the CAMSAP1-MT group had a longer OS than those in the CAMSAP1-WT group in both the Zhujiang cohort (HR = 0.46, 95% Cl 0.22–0.96, *p* = 0.036) and the reported cohort (HR = 0.23, 95% Cl 0.06–0.93, *p* = 0.024) (Figure 2D). The relationship among CAMSAP1 mutation, CAMSAP1 expression and OS had no statistical significance (Supplementary Figure S1).

### Relationship Between CAMSAP1 Status, Other Gene Mutations, and Clinical Characteristics

**FIGURE 3:**
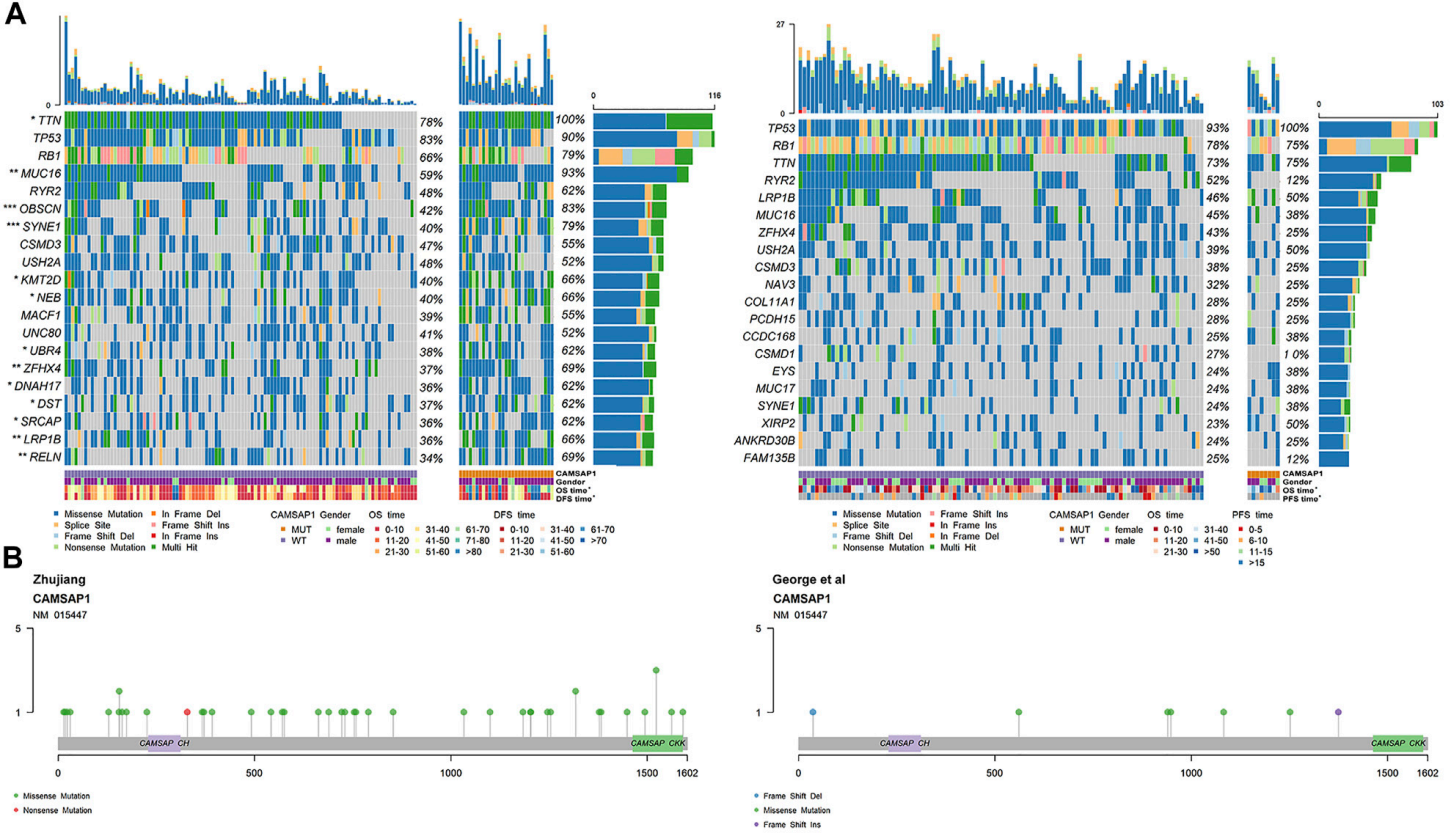
Genetic characteristics of SCLC patients. **(A)** Panoramic views of the 20 most frequently mutated genes in the Zhujiang and reported cohorts. Genes are sorted by mutation frequency. CAMSAP1 status, sex, OS, PFS, mutation type and mutation frequency are marked. ***: *p* < 0.001; **: *p* < 0.01; *: *p* < 0.05. **(B)** Lollipop plot showing the distribution of CAMSAP1 mutation in the Zhujiang and reported cohorts.

Among the genetic landscape of 137 samples from the Zhujiang cohort and 108 samples from the reported cohort (Figure 3A), TP53, RB1, TTN, RYR2, MUC16, SYNE1, CSMD3, USH2A, ZFHX4 and LRP1B were the top 20 most frequently mutated genes. In the Zhujiang cohort, the mutation frequency of the following genes was higher in the CAMSAP1-MT group than in the CAMSAP1-WT group; TTN (100 vs. 78%), MUC16 (93 vs. 59%), SYNE1 (79 vs. 40%), ZFHX4 (69 vs. 37%), LRP1B (66 vs. 36%). Missense mutation was the main mutation type of the following: TP53, TTN, RYR2, MUC16, SYNE1, CSMD3, USH2A, ZFHX4 and LRP1B. The RB1 mutations were predominantly splice site and nonsense mutations. In addition, we compared the clinical features (including gender, PFS and OS) of the CAMSAP1-MT group and CAMSAP1-WT group. In Zhujiang cohort, patients in the CAMSAP1-MT group showed a longer OS and PFS than those in the CAMSAP1-WT group. In the reported cohort, only OS in the CAMSAP1-MT group was longer. In both Zhujiang and reported cohorts, there was no significant difference in gender between the two groups. Figure 3B shows the main mutation sites of CAMSAP1 in both cohorts. The main mutation type can be seen here as missense mutation and is located at CAMSAP CKK and CAMSAP CH domain.

### The Interaction Among Mutant Genes and the Relationship Between CAMSAP1 Status and Oncogene Status

**FIGURE 4:**
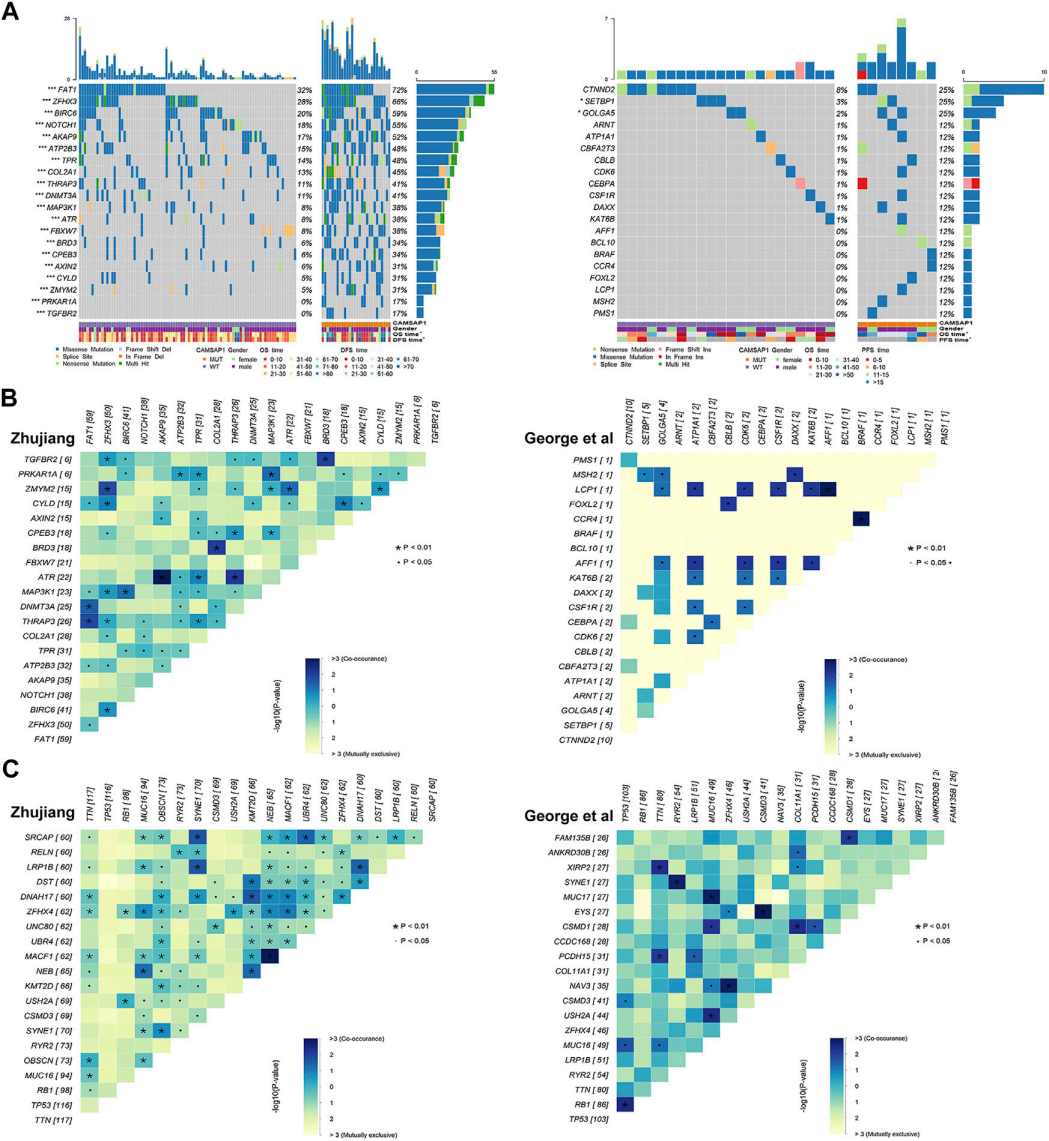
Oncogene mutations in SCLC patients. **(A)** Panoramic views of the 20 most frequently mutated oncogenes in the Zhujiang and reported cohorts. Genes are sorted by mutation frequency. CAMSAP1 status, sex, OS, PFS, mutation type and mutation frequency are marked. ***: *p* < 0.001; **: *p* < 0.01; *: *p* < 0.05. **(B)** Co-occurrence and mutual exclusivity among the 20 most frequently mutated oncogenes in the Zhujiang and reported cohorts. **(C)** Co-occurrence and mutual exclusivity among the 20 most frequently mutated genes in the Zhujiang and reported cohorts.

In order to investigate the relationship between CAMSAP1 mutation and oncogene mutations in the two cohorts, we used WES and clinical data in combination with the NCG database to compare the oncogene characteristics in the CAMSAP1-MT and CAMSAP1-WT groups. In the landscape of the Zhujiang cohort (Figure 4A), the mutation frequency of oncogenes in the CAMSAP1-MT group was significantly higher than that in the CAMSAP1-WT group (*p* < 0.05). In the CAMSAP1-MT group, the oncogenes with a mutation frequency >50% were FAT1 (72 vs. 32%), ZHFX3 (66 vs. 28%), BIRC 6 (59 vs. 20%), NOTCH1 (55 vs. 18%) and AKAP6 (52 vs. 17%). The most common mutation type among these genes was missense mutation. In the reported cohort (Figure 4A), the oncogenes with the highest mutation frequency were CTNND2, SETBP1 and GOLGA5. For the reported cohort, the mutation frequency in the CAMSAP1-MT group was significantly higher than that in the CAMSAP1-WT group for SETBP1 (25 vs. 3%) and GOLGA5 (25 vs. 2%). The mutation frequency of other oncogenes was not significantly different between the two groups. In the Zhujiang cohort, ZHFX3 mutation was concurrent with BIRC6 mutation (*p* < 0.01) but mutually exclusive with FAT1 (*p* < 0.05). In the reported cohort, all oncogenes with statistical significance were concurrent (Figure 4B).

We also performed a co-occurrence and mutual exclusivity analysis among all gene mutations (Figure 4C). Among the common gene mutations in both the Zhujiang and reported cohorts, SYNE1 and RYR2, USH2A and MUC16, and MUC16 and TTN were concurrent in the Zhujiang cohort but mutually exclusive in the reported cohort.

### Transcriptome Characteristics Related to CAMSAP1 Mutation

In both cohorts, we compared the expression data between the CAMSAP1-MT and CAMSAP1-WT groups by using the R package “limma” to identify the DEGs. In the Zhujiang cohort, compared with the expression in CAMSAP1-WT group, there were 830 up-regulated genes and 496 down-regulated genes in the CAMSAP1-MT group. In the reported cohort, compared with the expression in CAMSAP1-WT group, there were 250 up-regulated genes and 175 down-regulated genes in the CAMSAP1-MT group (Supplementary Figure S2A). The expression of top 20 genes with the largest LogFC in the CAMSAP1-MT group and CAMSAP1-WT group is shown in Supplementary Figure S2B.

**FIGURE 5:**
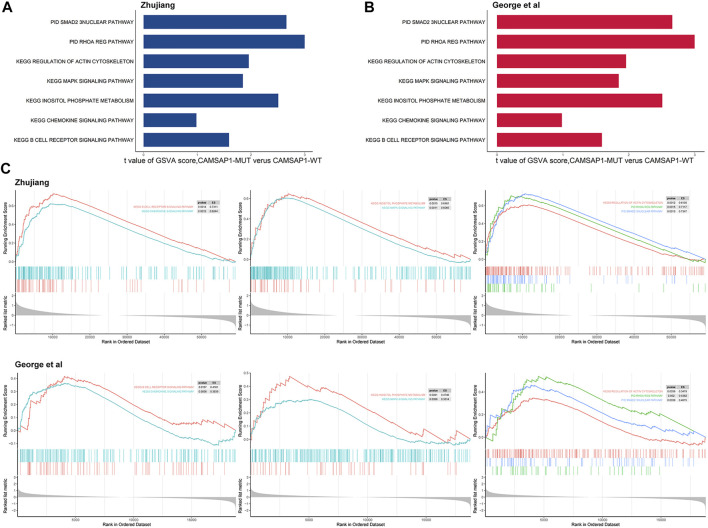
Transcriptome functional characteristics of the CAMSAP1-MT and the CAMSAP1-WT groups. **(A)** The difference in GSVA scores between the CAMSAP1-MT and CAMSAP1-WT groups in the Zhujiang cohort. **(B)** The difference in GSVA scores between the CAMSAP1-MT and CAMSAP1-WT groups in the reported cohort. **(C)** GSEA results of platinum drug sensitivity and prognosis related pathways, including anti-tumor immune pathway, platinum-mediated apoptosis pathway and cell activity pathway. The results above reflect the Zhujiang cohort, and the results below reflect the reported cohort. Yellow represents mutual exclusivity and green represents co-occurrence. *: *p* < 0.01.

In order to determine the mechanism underlying the improved prognosis in the CASAP1-MUT group, GSVA and Kaplan-Meier analyses were used. In the CAMSAP1-MT group, GSVA scores of anti-tumor immunity, platinum-mediated apoptosis, and cell activity pathways were significantly higher than those in the CAMSAP1-WT group, and patients with high GSVA scores showed better prognosis than those with low GSVA scores (Figures 5A,B). For verification, we used the previously identified DEGs to carry out GSEA analysis. The results were consistent, also showing that anti-tumor immunity, platinum-mediated apoptosis and cell activity pathways were significantly up-regulated in CAMSAP1-MT groups in the two cohorts (Figure 5C).

### Potential Therapeutic Drugs and Mode of Action Based on CAMSAP1 Mutation

**FIGURE 6:**
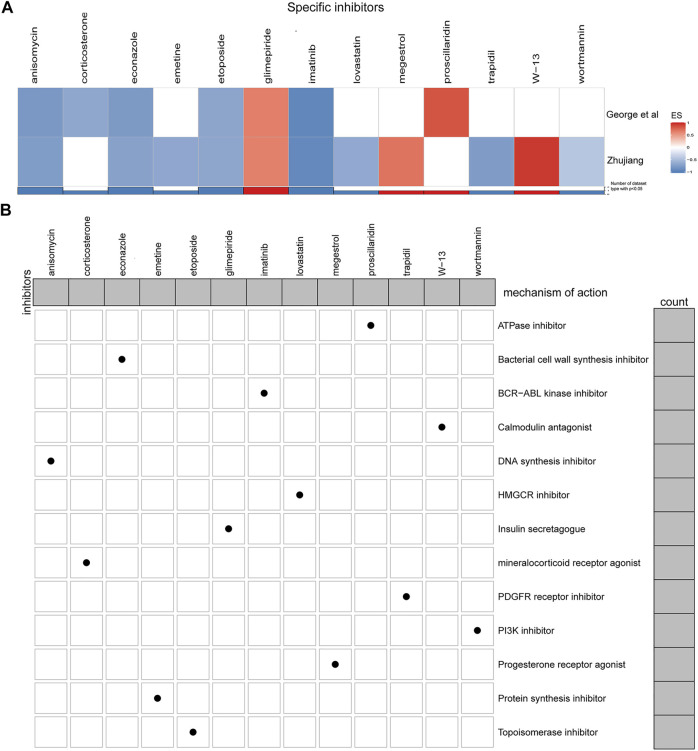
Potential CAMSAP1 targeted drugs. **(A)** Heatmap showing the enrichment scores of each compound obtained by cMAP analysis in the Zhujiang and reported cohorts. Red indicates an enrichment score >0 and blue indicates an enrichment score <0. **(B)** Heatmap showing the MoA of each compound. The histogram on the right shows the number of compounds in each MoA. The histogram on the top shows the number of MoA of each compound. MoA: Mode-of-action.

We utilized the cMAP database, inputting up- and down-regulated genes, and searched for potential drugs that target CAMSAP1. Eleven of the known tumor therapeutic drugs were enriched in the Zhujiang cohort, and seven in the reported cohort (*p* < 0.05). There were five drugs that were enriched in both cohorts, and thus, identified as being potential therapeutic drugs for SCLC patients with CAMSAP1 mutation. These were: anisomycin, econazole, etoposide, glimepiride and imatinib (Figure 6A). MoA is a group of drugs with similar gene markers and therapeutic effects (Ma et al., 2013). Through cMap MoA analysis, we found that 13 drugs were enriched in 13 modes of action (Figure 6B).

## Discussion

Etoposide combined with cisplatin (EP) or irinotecan combined with cisplatin (IP) are the first-line treatments for SCLC patients (Ogino et al., 2016), and the overall response rate (ORR) is 57% (Rossi et al., 2012). However, most patients will develop resistance to these chemotherapy drugs and eventually die of recurrent diseases (Jett et al., 2013). Therefore, it is of great importance to find predictive markers which can be used to predict and improve platinum drug sensitivity in order to improve the prognosis of SCLC patients. Previous studies have shown that specific gene mutations are associated with higher platinum drug sensitivity in many cancers, such as CCDC69 mutation in ovarian cancer, ERCC2 mutation in bladder cancer, and BRCA mutation in pancreatic cancer (Lohse et al., 2015; Cui et al., 2019; Li Q. et al., 2019). Therefore, in this study we attempted to discover the gene markers related to the increased sensitivity of platinum drugs in patients with SCLC. We analyzed WES and clinical data from a reported SCLC cohort (reported by George et al. (2015) and an SCLC cohort from Zhujiang Hospital and Sun Yat-sen University Cancer Center. We found a significant relationship between CAMSAP1 mutation and both increased sensitivity to platinum drugs and the improved prognosis of SCLC patients treated with platinum-based chemotherapy.

Our analysis of oncogenes showed that the mutation frequency of some such as ZHFX3, BIRC6, Axin2, CPEB3, TPR, Axin2, Notch1, DNMT3A and COL2A1 was significantly higher in the CAMSAP1-MT group compared to the CAMSAP1-WT group, and that this was closely related to improved prognosis. ZHFX3, BIRC6, Axin2, CPEB3 and TPR can regulate the proliferation and apoptosis of tumor cells. After silencing CPEB3, the growth of tumor cells increases while apoptosis decreases (Lin et al., 2019). Thus, CPEB3 mutation may inhibit the growth of tumor cells by increasing CPEB3 expression. ZHFX3 and BIRC6 can promote the proliferation of tumor cells, meaning that the mutation or knockout of these can inhibit the progression of cancer, resulting in a prolonged OS (Hao et al., 2020; Zhang et al., 2021). Of note, BIRC6 and ZHFX3 mutations always appear together (Figure 4). Silencing of TPR can trigger G0-G1 block, mediate cell aging through p53 and promote apoptosis of tumor cells (David-Watine, 2011). Axin2 is a negative regulator of the Wnt/β-catenin pathway and, once activated, can inhibit the proliferation and formation of tumors by inhibiting Wnt/β-catenin (Yu et al., 2019). In addition, some Axin2 mutant genotypes are closely related to the low-risk lung cancer types (Kanzaki et al., 2006; Gunes et al., 2009). Notch1 and DNMT3A are not only related to the proliferation of tumor cells, but also to the regulation of invasion and metastasis, which can influence patient prognosis (Gan et al., 2018; Xiao et al., 2020). It has been shown that patients with high COL2A1 expression have delayed recurrence (Ganapathi et al., 2016). Due to the lack of consistency in oncogene mutations in the two cohorts, further verification is needed. This inconsistency may be caused by an insufficient number of samples and differences in patient ethnicity.

**FIGURE 7:**
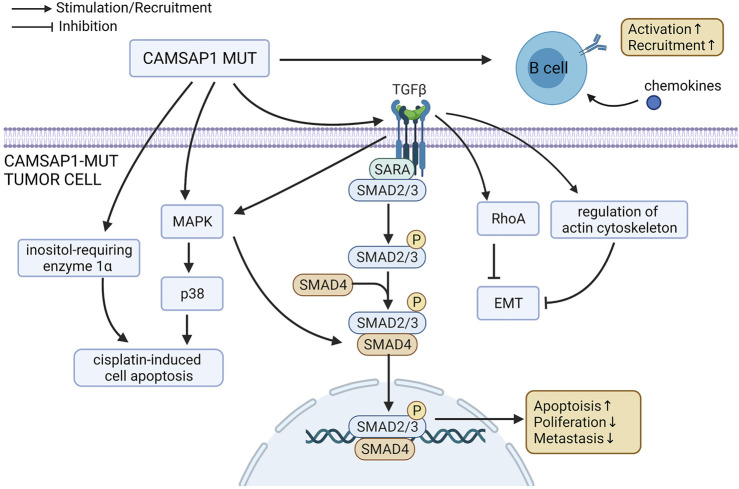
Possible mechanism underlying the improved platinum drug sensitivity and prognosis of CAMSAP1-MT SCLC patients.

Upon analysis of pathway enrichment, we found that a more favorable prognosis was related to the up-regulation of apoptosis-related pathway, anti-tumor immune pathway, and EMT inhibition pathway (Figure 7). Activation of inositol-requiring enzyme 1α (IRE1α) can enhance platinum-mediated cell death and affect patients’ drug sensitivity to platinum (Chen et al., 2019). P38 MAPK-Hsp27, which can be activated by platinum-based therapy, is another important pathway that regulates apoptosis and plays an important role in platinum-mediated cell death (Widmann et al., 1999; Chen et al., 2019). It has been shown that silencing the P38 protein results in a reduction of platinum-mediated apoptosis (Al-Khayal et al., 2020). Smad2/3 can be activated as result of the activation of TGF receptor by TGF-β, forming a complex with Smad4 (Derynck and Zhang, 2003). This is an important pathway affecting cell proliferation, differentiation and apoptosis, with the inhibition of Smad2 being shown to promote tumor metastasis (Ying et al., 2017). The expression of phosphorylated Smad2 (p-Smad2) is consistent with that of Smad4 in that a decrease in expression results in platinum resistance and poor prognosis (Huang et al., 2015; Liu et al., 2020; Wang et al., 2021). In both cases, this is due to the regulation of cell proliferation, adhesion and immune response. Therefore, the enrichment and up-regulation of the Smad2/3 nuclear pathway may improve patient prognosis and increase platinum drug sensitivity. Crosstalk mechanisms exist between the MAPK pathway and Smad pathways. TGF-β can activate P38 MAPK pathway, and the activated MAPK can then activate Smad through direct phosphorylation or effector molecules, ultimately leading to an increase in platinum drug sensitivity (Javelaud and Mauviel, 2005). Rho protein family is an important group of molecules that regulate cell morphology, movement, adhesion and proliferation, as well as the actin cytoskeleton (Steichen et al., 2021). The activation of RhoA can reduce the mobility of post-EMT cells, while the inhibition of RhoA can lead to a shortened PFS and increase in lymphatic metastasis (Bellovin et al., 2005). Many other actin cytoskeleton regulators have also been found to inhibit EMT, such as lovastatin (Zheng et al., 2021), Rho GTPase activating protein 10 (ARHGAP10) (Lin et al., 2021) and Ras-related C3 botulinum toxin substrate 1B (RAC1B) (Ungefroren et al., 2020). Therefore, up-regulation of the actin cytoskeleton/RhoA regulation pathway may increase platinum drug sensitivity and improve prognosis by inhibiting EMT, reducing metastasis and regulating the number of cancer stem cells (Li et al., 2019). B cells also play an important role in anti-tumor immunity and are related to better prognosis (Tong et al., 2016; Cabrita et al., 2020; Helmink et al., 2020; Petitprez et al., 2020; Shen et al., 2020). Chemokines, such as CXCL-13 and CXCL-5, can cause B cells to aggregate at the tumor site (Hussain et al., 2021). The enhancement of chemokines and B cell immune activity may be another reason for the improved prognosis in the CAMSAP1-MT group.

Based on the analysis of cMAP datasets, anisomycin, econazole, etoposide, glimepiride and imatinib are the potential therapeutic drugs for SCLC patients with CAMSAP1 mutation. Anisomycin can inhibit angiogenesis, proliferation and invasion of tumor cells by blocking the PI3K/Akt pathway (Ushijima et al., 2021)and Notch1 pathway (Ye et al., 2019). Econazole induces cell apoptosis and inhibits cancer invasion through the elevated protein level of p53 (Choi et al., 2020). Imatinib reduces the phosphorylation of the PDGFRα/Akt axis, suppressing tumor cell growth and migration (Nayeem et al., 2021). Etoposide, as a Topoisomerase inhibitor, has been approved by FDA and is widely used in cancer therapy (Montecucco et al., 2015). Glimepiride inhibits tumor cell growth through activation of AMPK(Long et al., 2020).

It should be noted that this study has some limitations. First of all, we did not compare the differences in the predictive effect of CAMSAP1 on platinum between different ethnicities. Secondly, the mechanism of how CAMSAP1 mutation affects drug sensitivity lacks experimental verification. Thirdly, the clinical characters of the two cohorts are not completely same and the number of samples in this study is insufficient, so the results should be verified in a larger population. Fourthly, the relationship between CAMAP1 mutation and other treatments could be further verified.

## Conclusion

In this study, we showed that CAMSAP1 mutation can serve as a suitable biomarker of platinum drug sensitivity. We then went on to explore the possible mechanisms and found that CAMSAP1 mutation improves the sensitivity of platinum drugs and the prognosis of SCLC patients by regulating a variety of cellular activities. These include tumor cell growth, apoptosis, invasion, metastasis, anti-tumor immunity, and EMT. Our results suggest that gene mutation may be the molecular basis for the change in chemosensitivity. In addition, this study also provides important evidence for the guidance of treatment and clinical experimental design in SCLC and other types of tumors associated with CAMSAP1 mutation.

## Data Availability

The datasets presented in this study can be found in online repositories. The names of the repository/repositories and accession number(s) can be found in the article/Supplementary Material.

## Abbreviations

SCLC, small cell lung cancer; ES-SCLC, extended-stage SCLC; WES, whole exome sequencing; GSVA, gene set variation analysis; GSEA, gene set enrichment analysis; cMAP; Connectivity Map; EMT, epithelial-mesenchymal transition; MoA, mode-of-action; EP, Etoposide combined with cisplatin; IP, irinotecan combined with cisplatin; ORR, overall response rate; OS, overall survival; PFS, progression-free survival.
