# Supplementary material for: CAMSAP1 Mutation Correlates With Improved Prognosis in Small Cell Lung Cancer Patients Treated With Platinum-Based Chemotherapy
Source: Front Cell Dev Biol. 2022 Jan 11;9:770811. doi: 10.3389/fcell.2021.770811 (PMC8787262; doi:10.3389/fcell.2021.770811)
Supplement: Supplementary file 1 [file DataSheet2.PDF]

Supplementary Table 2. Clinical characteristics of the Zhujiang cohort.

	CAMSAP1 MUT	CAMSAP1 WT	p
n	29	109	
age (mean (SD))	56.90 (13.06)	61.84 (9.54)	0.024
sex = male (%)	25 (86.2)	89 (81.7)	0.764
T_stage (%)			0.069
1	10 (34.5)	32 (29.4)	
2	10 (34.5)	56 (51.4)	
3	9 (31.0)	15 (13.8)	
4	0 (0.0)	6 (5.5)	
N_stage (%)			0.824
0	17 (58.6)	61 (56.0)	
1	1 (3.4)	5 (4.6)	
2	11 (37.9)	40 (36.7)	
3	0 (0.0)	3 (2.8)	
M_stage = 1 (%)	1 (3.4)	2 (1.8)	1
UICC_stage (%)			0.855
I	12 (42.9)	39 (36.4)	
II	4 (14.3)	17 (15.9)	
III	11 (39.3)	49 (45.8)	
IV	1 (3.6)	2 (1.9)	
smoking = smoker (%)	25 (86.2)	80 (75.5)	0.327
