# Supplementary figures and images for: CAMSAP1 Mutation Correlates With Improved Prognosis in Small Cell Lung Cancer Patients Treated With Platinum-Based Chemotherapy
Source: Front Cell Dev Biol. 2022 Jan 11;9:770811. doi: 10.3389/fcell.2021.770811 (PMC8787262; doi:10.3389/fcell.2021.770811)

A

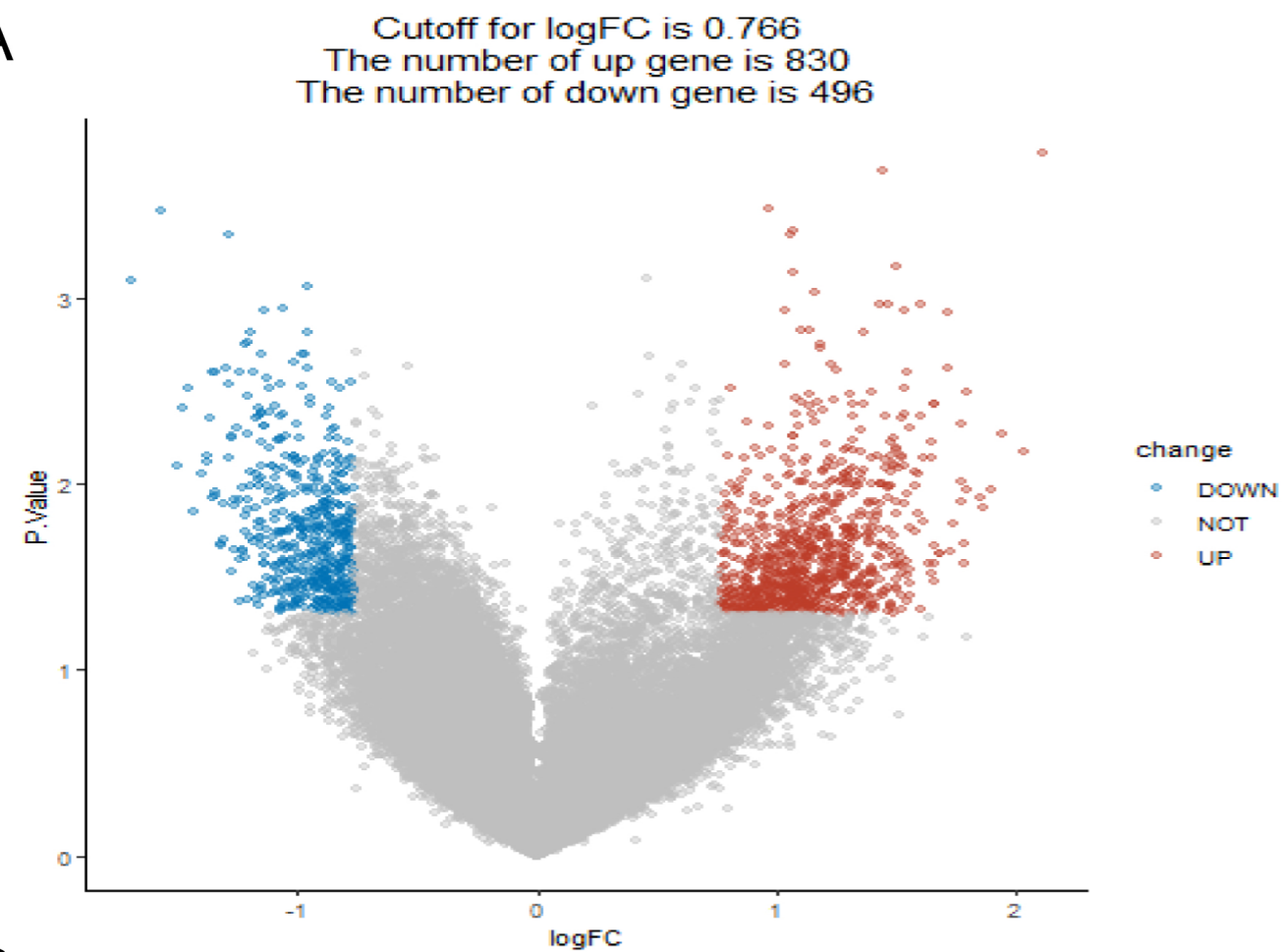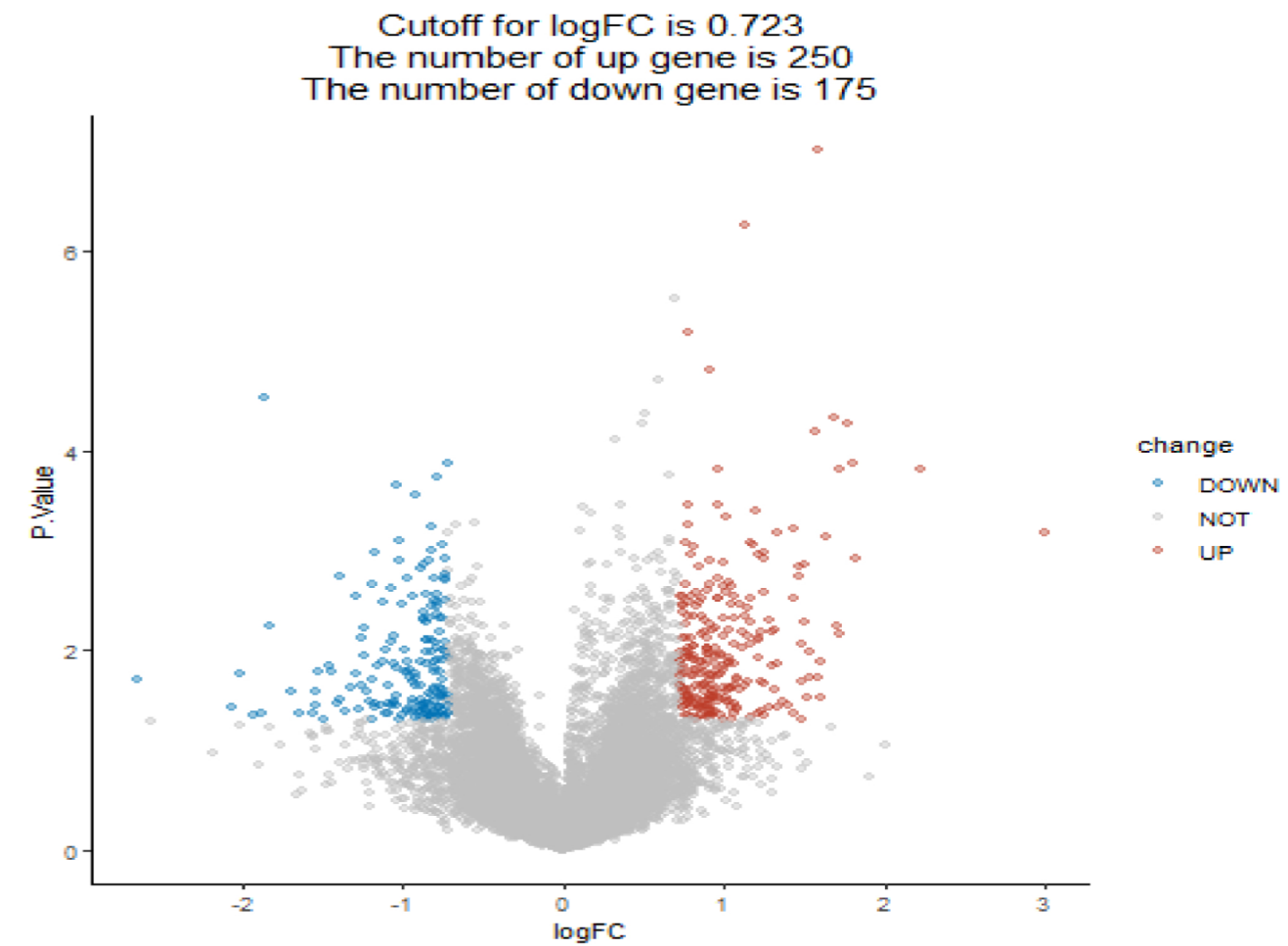

B

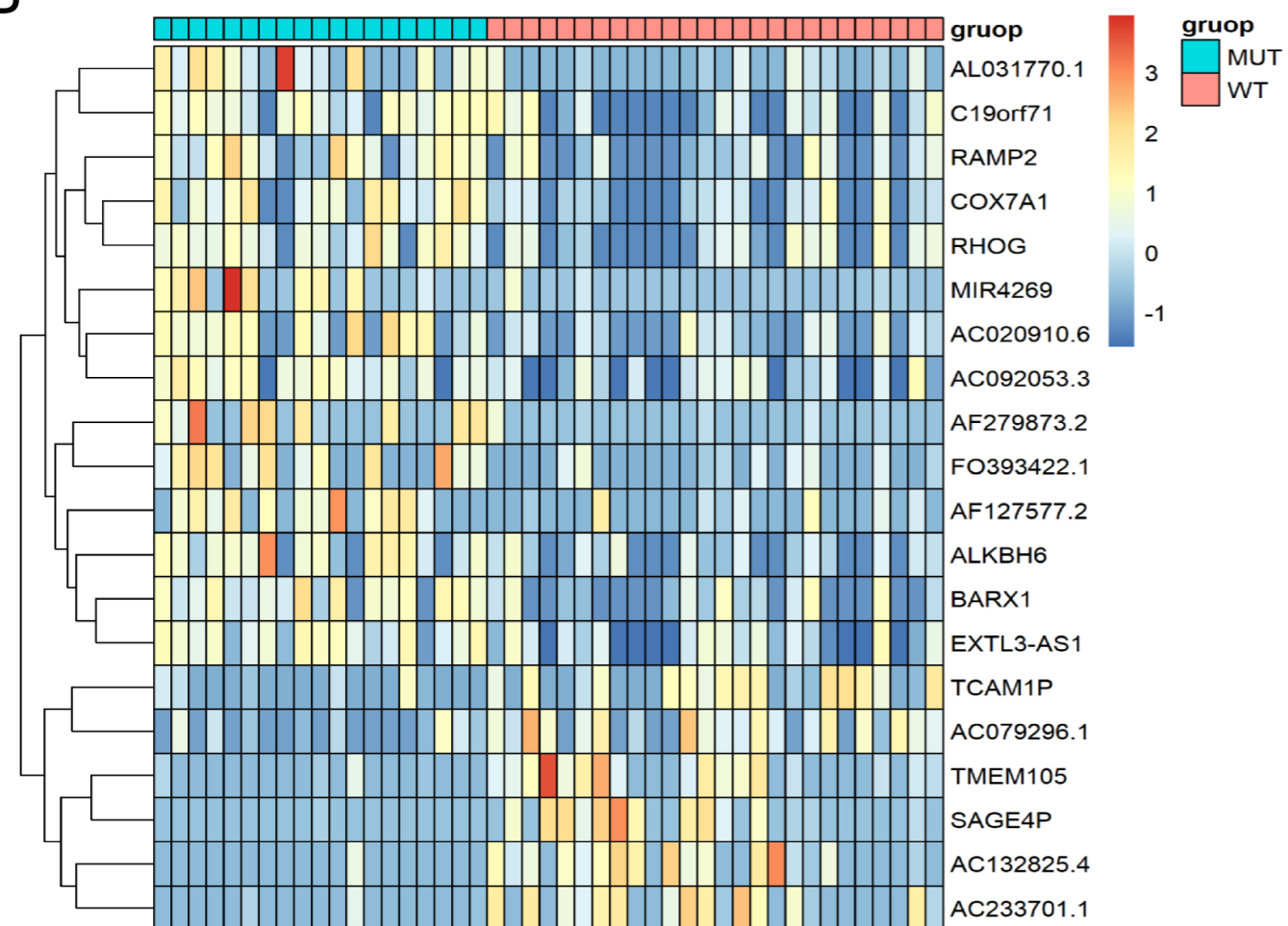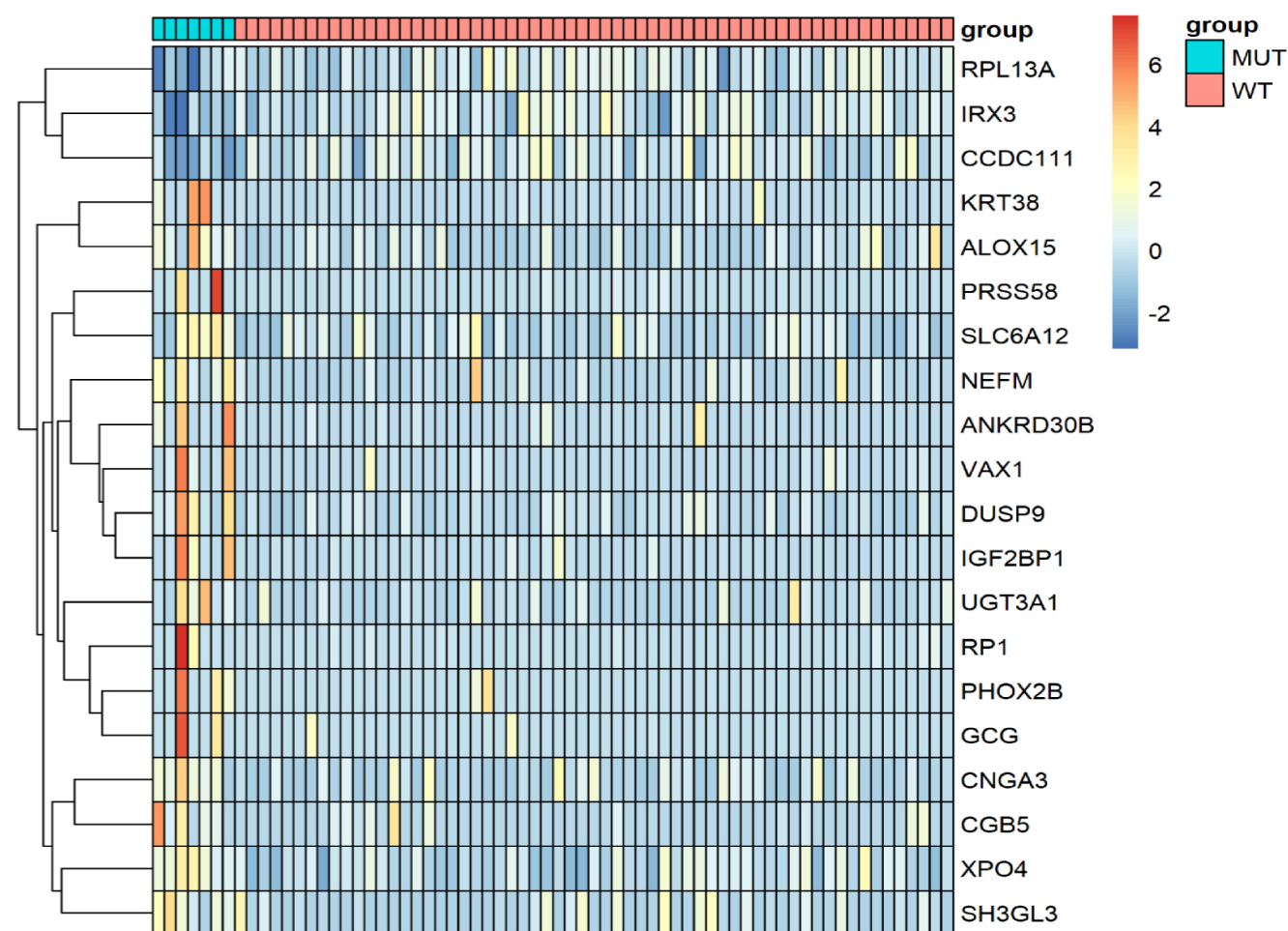

Supplement: Supplementary file 3 [file DataSheet4.PDF]

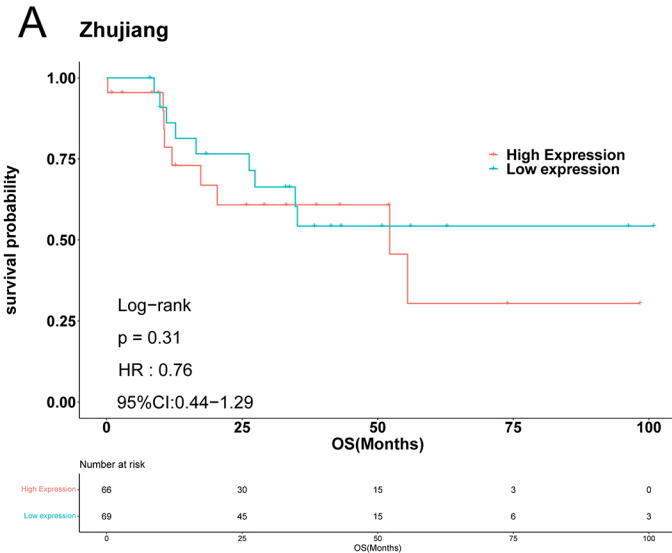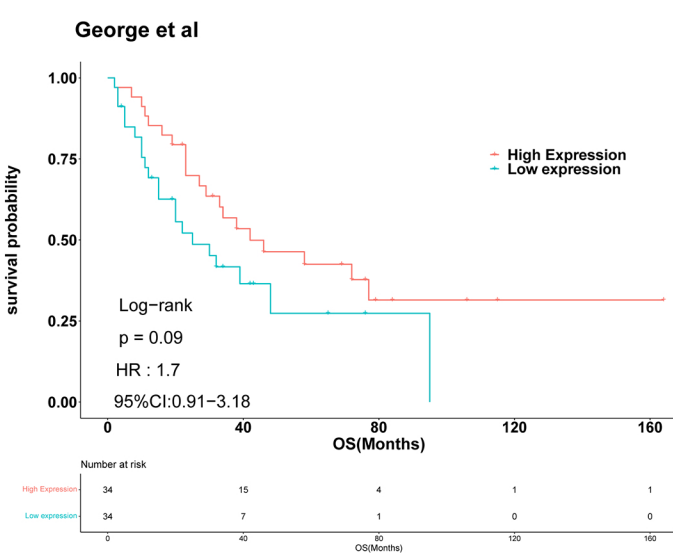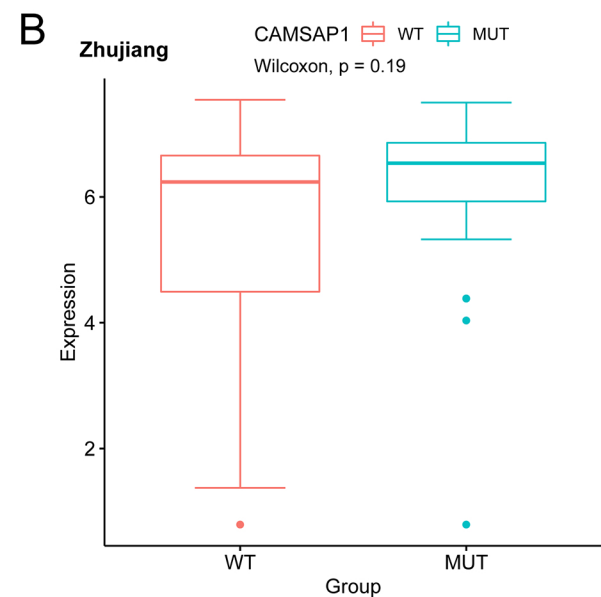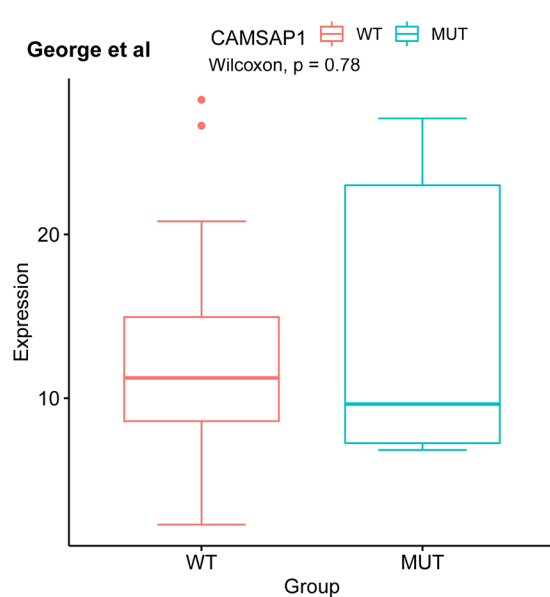

Supplement: Supplementary file 5 [file DataSheet3.PDF]
