# Supplementary material for: CAMSAP1 Mutation Correlates With Improved Prognosis in Small Cell Lung Cancer Patients Treated With Platinum-Based Chemotherapy
Source: Front Cell Dev Biol. 2022 Jan 11;9:770811. doi: 10.3389/fcell.2021.770811 (PMC8787262; doi:10.3389/fcell.2021.770811)
Supplement: Supplementary file 7 [file DataSheet1.PDF]

Supplementary Table 1. Clinical characteristics of the reported cohort.

	CAMSAP1 MUT	CAMSAP1 WT	p
n	8	93	
age (mean (SD))	63.75 (8.89)	64.78 (9.43)	0.765
sex = male (%)	5 (62.5)	59 (63.4)	1
T_stage (%)			0.56
1	4 (50.0)	27 (32.9)	
2	3 (37.5)	35 (42.7)	
3	0 (0.0)	13 (15.9)	
4	1 (12.5)	7 (8.5)	
N_stage (%)			0.714
0	4 (50.0)	34 (42.0)	
1	2 (25.0)	13 (16.0)	
2	2 (25.0)	26 (32.1)	
3	0 (0.0)	8 (9.9)	
M_stage = 1 (%)	0 (0.0)	18 (23.4)	0.336
UICC_stage (%)			0.492
I	4 (50.0)	30 (32.3)	
II	2 (25.0)	19 (20.4)	
III	2 (25.0)	25 (26.9)	
IV	0 (0.0)	19 (20.4)	
smoking = smoker (%)	8 (100.0)	84 (97.7)	1
